# Experience with intraoperative radiation therapy in an urban cancer center

**DOI:** 10.1186/s13014-023-02299-0

**Published:** 2023-07-25

**Authors:** Therese Youssef Andraos, Karin A. Skalina, Sheldon Feldman, Keyur Mehta, Wolfgang A. Tome, Maureen P. McEvoy, Anjuli M. Gupta, Jana L. Fox

**Affiliations:** 1 Department of Radiation Oncology, Montefiore Medical Center, Albert Einstein College of Medicine, Bronx, NY USA; 2 Department of Breast Surgical Oncology, Montefiore Medical Center, Albert Einstein College of Medicine, Bronx, NY USA

**Keywords:** Breast cancer, Intraoperative radiation therapy, Radiation therapy

## Abstract

**Background/objective:**

Intra-operative radiation therapy (IORT) is a newer partial breast irradiation technique that has been well studied in 2 large randomized trials, the TARGIT-A and ELIOT trials. We initiated our IORT program in 2018 in the context of a registry trial, and aim to report our early results thus far.

**Methods:**

We instituted an IORT practice using Intrabeam® low energy 50kVp x-rays for selected breast cancer cases in 2018. Patients were enrolled on our institutional registry protocol which allowed for IORT in ER + patients with grade 1–2 DCIS ≤ 2.5 cm or invasive disease ≤ 3.5 cm in patients of at least 45 years of age.

**Results:**

Between January 2018 and December 2021, 181 patients with clinical stage 0-IIA ER + breast cancer were evaluated. One hundred sixty-seven patients ultimately received IORT to 172 sites. The majority of patients received IORT at the time of initial diagnosis and surgery (160/167; 95.8%). Re-excision post IORT occurred in 16/167 patients (9.6%) due to positive margins. Adjuvant RT to the whole breast +/- LN was ultimately given to 23/167 (13.8%) patients mainly due to positive sentinel LN found on final pathology (12/23; 52%); other reasons were close margins for DCIS (3/23; 13%), tumor size (3/23; 4.3%), and multifactorial (5/23; 17.4%). Five patients (3%) had post-operative complications of wound dehiscence. There were 3 local recurrences (1.6%) at a median follow-up of 27.9 months (range: 0.7– 54.8 months).

**Conclusions:**

IORT has been proven to be a safe and patient-centered form of local adjuvant RT for our population, in whom compliance with a longer course of external beam radiation can be an issue. Long term efficacy remains to be evaluated through continued follow up. In the era of COVID-19 and beyond, IORT has been an increasingly attractive option, as it greatly minimizes toxicities and patient visits to the clinic.

**Trial registration:**

All patients were prospectively enrolled on an institutional review board-approved registry trial (IRB number: 2018–9409).

## Background

The local management of early breast cancer has evolved from radical mastectomy to lumpectomy with adjuvant radiation (RT). These two approaches have been demonstrated to yield equivalent rates of local control (LC) and overall survival (OS) [1]. Traditionally, radiation fields have included the entirety of the breast, with daily treatments spanning 3–6 weeks. Given that the majority of early in-breast recurrences occur near the site of the original primary tumor [2], efforts have been made in selected patients to limit the target to solely the portion of the breast surrounding the index lesion. This approach, known as Partial Breast Irradiation (PBI), leads to a greater ability to spare normal breast tissue, lung, and heart, and reduces the potential for toxicity.

Appropriate patients for PBI must be selected carefully. Several professional societies have provided published eligibility criteria for PBI, including the American Society of Breast Surgeons (ASBrS) [3], the American Brachytherapy Society (ABS) [4, 5], the National Surgical Adjuvant Breast and Bowel Project (NSABP)/the Radiation Therapy Oncology Group (RTOG) [6] and the American Society for Therapeutic Radiology and Oncology (ASTRO).

Intraoperative radiation therapy (IORT) is the newest accelerated PBI (APBI) technique that uses a targeted, single high-dose of RT in the operating room, performed concurrently with breast conserving surgery for low-risk patients. Two randomized control trials, TARGeted Intraoperative radioTherapy (TARGIT-A) and ELectron IntraOperative radioTherapy (ELIOT), have compared IORT to whole breast irradiation (WBI) in terms of LC and OS for low-risk patients. Long term follow up data of the ELIOT trial showed a higher rate of local recurrence in the IORT group (12.6% vs. 2.4% at 15 years) [7, 8]. The TARGIT-A trial looked at using low energy (50 kV) x-rays to a dose of 20 Gy prescribed to the surface of a spherical applicator with the Intrabeam® device. Their 5-year local recurrence rate (LRR) was reported as 2.11% with IORT versus 0.95% with WBI.

We report herein our experience using IORT with Intrabeam® located in one of the 5 boroughs of New York City with the highest ratio of medically underserved patient population.

## Methods

### Study design and participants

This is a single center, prospective, observational, institutional review board-approved registry trial (IRB number: 2018–9409) of patients treated from January 2018 to December 2021, designed to track LC rates and side effect profiles of IORT. Eligibility criteria include female patients, age ≥ 45 years, with either cT1-2N0, ≤ 3.5 cm estrogen receptor -positive (ER+) invasive breast cancer, or Grade 1–2 ER + ductal carcinoma in-situ ≤ 2.5 cm, mammographically detected. Patients should be suitable for breast conserving surgery (BCS) and have no contraindication to radiation. Patients with a history of ipsilateral cancer and/or prior in-field RT are also eligible. Clinically axillary node positive patients were excluded, as were patients with multicentric disease, BRCA 1 or 2 genetic mutations, and those undergoing neoadjuvant chemotherapy.

### Treatment technique

Following BCS, patients deemed eligible for IORT and who enrolled on the registry trial received a single dose of RT of 20 Gy to the lumpectomy cavity. The IORT technique used in our protocol is with the Intrabeam® system which utilizes a miniature electron beam-driven 50 kV x-ray source at the tip of a 3.2 mm diameter tube. The radiation source can be inserted into the surgical cavity immediately after tumor removal, providing intraoperative radiotherapy directly to the tissues at the highest risk of recurrence. The Intrabeam® device is FDA approved for use in any part of the body. For breast irradiation, the radiation source is covered by a spherical applicator which ranges in diameter from 1.5 to 5 cm and conforms to the lumpectomy cavity. Surgical sutures are often used to maintain accurate conformance of the breast tissue to the applicator, while also ensuring protection of the skin and deeper structures. The X-ray source is small and lightweight and is mounted on a surgical arm and balanced for ease of delivery and support during treatment. The low energy of the radiation is easily shielded by sterile sheet shielding material applied around the irradiated area. In conjunction with distance, this is typically sufficient to reduce the dose below the regulatory limit for non-badged personnel outside of an unshielded operating room. Radiation levels inside and outside the operating room are measured by the physics team to ensure that they are below regulatory limits. During the radiation delivery, all personnel vacate the operating room for safety. The dose is prescribed to the surface of the applicator, and it rapidly attenuates to 5–7 Gy at 1 cm depth. Radiation is delivered over 15–45 min to the tumor bed, depending on the diameter of the applicator required to best fit the cavity. Appropriate applicator size was determined intraoperatively - upon agreement between the breast surgeon and the treating radiation oncologist - in terms of apposition to adjacent breast tissue without intervening air pockets and a goal of 1 cm of distance to overlying skin as seen on ultrasound.

Additional whole breast RT using 3 dimensional conformal RT was delivered to patients found to have high-risk unfavorable features on post-operative pathology, such as involved lymph nodes, larger tumor sizes, involved margins and/or high grade DCIS. In these patients, IORT functioned as a lumpectomy cavity boost.

The primary outcome was LC. Secondary outcomes included patterns of ipsilateral breast tumor and regional recurrences and toxicity rates.

### Statistical analyses

Baseline patient and tumor characteristics, including age, race, ethnicity, body mass index, ER/PR status, T stage, grade, and histology were collected. IORT applicator size and closest skin bridge were also recorded. Margin status, lymphovascular invasion (LVI) and lymph node status were extracted from the final pathology report. Side effects including wound healing issues were also captured. Continuous variables were expressed using sample medians, while categorical variables were expressed as percentages. Descriptive statistics tests were performed using IBM SPSS Statistics 27.0 (IBM Corporation, Armonk, NY, USA).

## Results

### Patient, tmor, and treatment characteristics

One hundred and eighty-one patients with clinical stage 0-IIA ER + breast cancer were referred for consideration for IORT. One hundred sixty-seven patients ultimately received IORT to 172 sites. Pre-operative MRI was obtained in 50.3% of the patients (91/181), which changed management and precluded IORT in 3 (3.3%). Reasons for not proceeding with IORT were radiographic and pathologic findings of additional lesions (3/14; 21.4**%**), patient preference (3/14; 21.4%), technical issues (3/14; 21.4%), and patient lost to follow-up (5/14; 35.7%). Baseline characteristics are summarized in Table 1. Fifteen patients were treated with neoadjuvant endocrine therapy.

**Table 1:** Baseline demographic and tumor characteristics of our patient cohort who received IORT (N = 167)

Baseline characteristics	N (%)
Age, median (range), yrs	66 (49–89)
45–49 yrs	1 (0.6%)
50–59 yrs	40 (24%)
60–69 yrs	76 (45.5%)
70–74 yrs	35 (21%)
>74 yrs	15 (9%)
Ethnicity	
Hispanic or Latino	65 (38.9%)
Not Hispanic or Latino	76 (45.5%)
N/A	26 (15.6%)
Race	
Black or African American	41 (24.6%)
White	39 (23.4%)
Asian	3 (1.8%)
Other Pacific Islander	1 (0.6%)
Other	63 (37.7%)
N/A	20 (12%)
Initial Pathology per lesion (N = 172)	
Invasive ductal carcinoma	120 (69.8%)
Invasive lobular carcinoma	9 (5.2%)
Ductal carcinoma in-situ	39 (22.7%)
Other	4 (2.3%)
Receptor status per lesion (N = 172)	
ER positive	172 (100%)
PR positive	154 (89.5%)
Her-2/neu positive	6 (3.5%)
Clinical tumor size, median (range), mm	10 (1–40)
Clinical Stage per lesion (N = 172)	
0	40 (23.3%)
IA	118 (68.6%)
IB	5 (2.9%)
IIA	9 (5.2%)
Depth from nipple, median (range), cm	6 (1–19)
BMI, median (range), kg/m^2^	29.3 (19.3–60.8)

The majority of patients received IORT at the time of initial surgery (160/167; 95.8%). Three patients (1.8%) received IORT at the time of re-excision and 4 (2.4%) received IORT at the time of recurrence or development of a new ipsilateral primary. Of those who received IORT on recurrence, 2 patients (50%) had previous RT to the ipsilateral breast. Table 2 summarizes the IORT applicator size and closest skin bridge distance.

**Table 2:** Treatment characteristics received by IORT patients

Treatment characteristics	N (%)
IORT to bilateral breasts	4 (2.4%)
IORT applicator size per lesion, cm (N = 172)	
3	46 (26.7%)
3.5	72 (41.9%)
4	39 (22.7%)
4.5	8 (4.7%)
5	7 (4.1%)
Closest skin bridge, median (range), mm	14 (7– 27.8)
Skin dose at closest margin, median (range), Gy	1.3 (0.2– 9.1)

Among patients who received IORT, lymph nodes (LN) were sampled in 133/136 (97.8%) patients with invasive disease and 9/31 (29%) of patients with DCIS. Eighteen patients with invasive cancer (13.5%) had some degree of LN involvement on final pathology, of which 8 had macrometastases, 6 had micrometastases, and 4 had isolated tumor cells. The median number of involved LNs was 1 (range, 1–5). Final pathology status is summarized in Table 3.

**Table 3:** Result on final pathology post-lumpectomy and IORT

Final pathology status	N (%)
Grade on final pathology per lesion (N = 172)	
1	39 (22.7%)
2	111 (64.5%)
3	20 (11.6%)
NA	2 (1.2%)
Lymphovascular invasion	15 (8.7%)
Positive LN status per patient (N = 167)	18 (10.8%)
Macroscopic disease	8 (44.4%)
Microscopic disease	6 (33.3%)
Isolated tumor cells	4 (22.2%)

Re-excision after IORT occurred in 16 patients (9.6%). Adjuvant RT to the whole breast +/- LN was ultimately given to 23/167 (13.8%) patients mainly due to positive sentinel LN found on final pathology (12/23; 52%); other reasons were close margins for DCIS (3/23; 13%), tumor size (3/23; 4.3%), and multifactorial (5/23; 17.4%). One patient with micrometastases was recommended to have additional radiation but did not return and was lost to follow up and another with N2a disease refused all adjuvant therapy.

### Clinical outcomes

Patients were followed clinically and radiographically, with clinical follow-up beginning 2 weeks post-operatively. Thirty-six patients had a BIRADS3 or 4 ipsilateral mammogram reading after surgery and IORT. Twelve patients underwent biopsy based on abnormal mammogram results; 3 of those had local recurrences. The remaining 9 were benign pathology.

Median follow-up was 27.9 months (range: 0.7– 54.8 months) for our surviving patients, with 3 local recurrences found (1.8%) at a median of 22.9 months. 1 patient passed away due to sepsis in the setting of metastatic colon cancer and another passed away due to COVID-19 complications, yielding an OS rate of 98.8%.

One of the local recurrences occurred in a patient with initially a grade 2 DCIS, ER+/PR- in 3 foci, the largest measuring 6.5 mm, resected with a margin of 2 mm. The diagnostic mammogram had noted calcifications spanning 4 cm, but biopsies of the 1:00 position revealed DCIS while those of the 2:00 position revealed atypical ductal hyperplasia only. Breast MRI did not reveal additional suspicious areas of disease. She recurred with invasive lobular carcinoma picked up on mammogram as a 5 mm nodule in the same quadrant just under 2 years later. She was treated with repeat lumpectomy and WBI.

Another LR was in a 69-year-old patient with a history of ER-negative DCIS treated previously with BCS and RT and then presented 6 years later with a contralateral 4 mm ER + grade 1 invasive ductal carcinoma (IDC). She underwent lumpectomy with IORT and recurred with a Stage I triple negative breast cancer in a different quadrant just under 2 years later. She was treated with repeat lumpectomy, adjuvant chemotherapy and WBI.

The third recurrence was in a 64-year-old woman bridged with anastrozole upon being diagnosed with a T1N0 well differentiated ER + IDC in the right lower inner quadrant at the beginning of the COVID-19 pandemic in March 2020. She ultimately underwent lumpectomy, sentinel node biopsy and IORT in June 2020 and was found to have a grade 1 pT1cN0 IDC. She resumed anastrozole and was found 2 years later to have a new nodule in the retroareolar region. She underwent lumpectomy and sentinel LN biopsy and was found to have pT1bN1mic grade 1 ER+/PR+/Her2- IDC again. She was treated with whole breast and regional nodal irradiation and switched to exemestane.

In terms of toxicity, 5 patients (3%) had post-op complications of wound dehiscence, including one case of full thickness skin necrosis. Chart review revealed 4 patients (2.4%) with documentation of lymphedema of the breast and/or arm after surgery and IORT, prompting referral for physical therapy. All of these patients had sentinel lymph node biopsies; one of which had whole breast radiation and lymphedema was localized to the breast only.

## Discussion

This cohort of patients had a 98.2% rate of locoregional control at a median follow-up of 27.9 months in our underrepresented minority population. Our results highlight the efficacy and feasibility of IORT in an underserved community. Our cohort is unique as 39% of our patients identified themselves as Hispanic or Latino, and more than 70% identified as non-white. It is important to emphasize that the 2 landmark trials did not provide a breakdown of the ethnic/racial subgroups in their trial. It is also worth noting that racial disparities have been correlated with worse prognosis especially in Black patients with prostate cancer [9], as well as breast cancer [10].

IORT has been compared to conventional WBI in the ELIOT and TARGIT-A studies [11]. The ELIOT trial was a randomized controlled equivalence trial which randomized 1305 women with breast cancer aged 48–75 years with tumors ≤ 2.5 cm to receive either IORT or standard WBI after lumpectomy. The IORT treatment was performed using electrons with 6 to 9 MeV energies to a dose of 21 Gy. Notably, additional adjuvant RT to the lymph nodes was only given in patients with ≥ 4 axillary lymph nodes positive in either arm [8]. The 5-year risk of ipsilateral breast tumor recurrence (IBTR) was 4.4% with IORT versus 0.4% with WBI (p = .0001), and 5-year survival rates were similar between the two groups. In a recent update on the long-term recurrence and survival outcomes of the ELIOT trial, the higher rate of IBTR in the IORT group persisted: 10- and 15-year IBTR rates were 8.1%, and 12.6% respectively in the IORT group compared to 1.1%, and 2.4% in the WBI arm [7]. However, the higher IBTR rates did not translate into any differences in OS. The authors report an unplanned analysis in which they identified a subgroup of 10.8% of their study population with a very low risk of recurrence (1.3% at 10 years). These are patients with Luminal A tumors less than 1 cm with a Ki-67 of less than 14%. While they state that this group can be safely considered for IORT, they do not state what treatment these patients received on their trial. Amongst patients considered “suitable” for PBI per the ASTRO guidelines, local recurrence rates with IORT alone were at 2%, 6.1% and 13.1% at 5, 10 and 15 years, and the authors suggest stricter criteria should be used when selecting patients for this modality.

TARGIT-A was another clinical trial comparing IORT to WBI, utilizing the same radiation technique as in our cohort. This study was a non-inferiority trial which randomized 3451 lumpectomy patients to receive IORT with Intrabeam® device or standard WBI. Eligibility criteria included women aged 45 years or more with early-stage clinically node negative ER + invasive ductal carcinoma undergoing BCS. Patients could receive IORT either at the time of lumpectomy or as an additional procedure after pathology was returned from the first surgery. Adjuvant WBI was needed in 15.2% of patients following IORT due to the final pathology showing positive lymph nodes, positive surgical margins, or high-risk tumor biology. At a median follow-up of 8.6 years the 5-year IBTR rates were 2.11% for IORT versus 0.95% for WBI, which was within the prespecified non-inferiority threshold of an absolute difference of 2.5% [12]. There was no significant difference in the overall survival rates between both arms. An additional analysis of this cohort of patients published by Vaidya et al. proposed the possibility of an abscopal effect in patients receiving IORT during lumpectomy [13].

There are important differences to note between the ELIOT and TARGIT-A trials. They used different IORT techniques, with electrons in the ELIOT and low-energy x-rays in TARGIT-A. As described by Vaidya et al. [14]., the degree of dissection of breast tissue required in order to deliver electron beam IORT is much more extensive than with the Intrabeam® device, in order to place a metal shield at the chest wall and then aim an aperture at the breast tissue to deliver the electrons. This extensive dissection likely creates more tissue hypoxia which is known to reduce radiation sensitivity. With Intrabeam^®^, immediately after the lumpectomy is performed, the surrounding breast tissue is suture in direct apposition to the applicator that delivers the radiation, ensuring full dose to the target tissue in a way electron beam cannot. While longer follow up is required for the TARGIT-A trial, these differences in techniques may account for inferior local control with electron IORT.Women ≥ 70 years of age comprise 30% of our cohort. While the CALGB 9343 and PRIME II trial [15–17] offer the option of no RT for those patients despite the increased - albeit still low risk of local failure, our study shows that IORT could be an alternative option for this group of patients who want the benefit of adjuvant RT without the added inconvenience of daily visits. This is currently being explored in the phase II prospective TARGIT-E trial [18].

23% of the patients in our cohort had clinical low-risk DCIS, one of whom had multiple foci and recurred with IORT alone. This subgroup of patients was not included in either of the landmark trials. In a prospective non-randomized trial looking at the role of IORT in patients with pure DCIS ≤ 4 cm in largest diameter which were deemed resectable with clear surgical margins after BCS, the study found that IORT was well tolerated with local recurrence rate of 5.7% at a median follow-up of 3 years [19]. As such, our study provides evidence of early acceptable outcomes for selected patients with DCIS treated with IORT.

Consistent with other published data, review of follow up notes revealed that our patients experienced very low rates of toxicity. Rates of lymphedema and/or wound dehiscence did not exceed 3%. In a recent paper on patient-reported outcomes with IORT versus WBI, patients reported better post-operative physical well-being of the chest, but there was no difference in patient-reported post-operative satisfaction with breast cosmesis or adverse effects of radiation [20]. Our study was more conservative in its inclusion of DCIS patients, limiting to those who fit the ASTRO consensus criteria for partial breast irradiation, namely ER + DCIS ≤ 2.5 cm. Our results provide evidence of early acceptable outcomes for carefully selected patients with DCIS treated with IORT.

Beyond the benefits of reduced radiation exposure to normal tissues, IORT has the advantage of being cost effective, both in terms of health care dollars and patients’ financial toxicity [21, 22]. This modality, when applied appropriately, eliminates the need for daily radiation treatments over the course of days to weeks, for which many patients present financial concerns surrounding transportation needs and missed work. Amongst our specific patient population of largely underserved minorities receiving concurrent chemoradiation, a recent study found a baseline rate of financial toxicity of 52%, prior to the initiation of any therapies. This rate increased by at least 25% over the course of treatments [23]. Another study found a correlation between financial toxicity and worse progression free survival amongst lung cancer patients [24]. Thus, not only can IORT help our healthcare delivery be more efficient, but it can also save our patients much needed and scarce resources.

Limitations of our study include short-term follow-up compared to the TARGIT and ELIOT trials and the non-randomized nature. Further follow-up is needed to determine the long-term IBTR, and OS rates of patients in our registry. The concept of IORT using low kV-X rays as a boost compared to an EBRT boost was studied retrospectively and IORT boost seems to yield similar local control rates as EBRT boost [25, 26]. This is being studied prospectively in the ongoing multicenter randomized controlled TARGIT-B trial (ClinicalTrials.gov Identifier: NCT01792726). Meanwhile, there are 2 large cohorts that showed acceptably low IBTR rates following IORT boost [27]. Thus, patients who go on to receive additional RT after surgery in our cohort arguably are still receiving a benefit from IORT in terms of time and cost savings.

## Conclusion

For women residing in underserved areas, IORT provides an appealing alternative option to daily external beam radiation for both early-stage breast cancer and DCIS. IORT has proven to be a safe and patient-centered form of local adjuvant RT for our patient population, in whom compliance with RT can be an issue. In the era of COVID-19, IORT was an increasingly attractive option, as it greatly minimized patient visits to the clinic. We will continue to follow these patients closely and report updated data as it evolves.

## Data Availability

The datasets used and/or analysed during the current study are available from the corresponding author on reasonable request.

## Abbreviations

ABS: American Brachytherapy Society
ASBr: American Society of Breast Surgeons
ASTRO: American Society for Therapeutic Radiology and Oncology
BCS: breast conserving surgery
cm: centimeter
DCIS: ductal carcinoma in situ
EBRT: external-beam radiation therapy
ELIOT: Electron IntraOperative radioTherapy
ER+: estrogen receptor positive
Gy: Gray
IBTR: ipsilateral breast tumor recurrence
IDC: invasive ductal carcinoma
IORT: intraoperative radiation therapy
LC: local control
LN: lymph node
LRR: local recurrence rate
LVI: lymphovascular invasion
MeV: megaelectronvolt
mm: millimeter
MRI: magnetic resonance imaging
NSABP: National Surgical Adjuvant Breast and Bowel Project
OS: overall survival
PBI: partial breast irradiation
RT: radiation therapy
RTOG: Radiation Therapy Oncology Group
TARGIT-A: Targeted Intraoperative radiotherapy
WBI: whole breast irradiation
